# Sex-Specific Differences in HLA Antibodies after Pneumococcal Vaccination in Kidney Transplant Recipients

**DOI:** 10.3390/vaccines7030084

**Published:** 2019-08-06

**Authors:** Monika Lindemann, Simon Oesterreich, Benjamin Wilde, Ute Eisenberger, Nils Muelling, Peter A. Horn, Falko M. Heinemann, Oliver Witzke

**Affiliations:** 1 Institute for Transfusion Medicine, University Hospital Essen, University Duisburg-Essen, 45147 Essen, Germany; 2 Department of Nephrology, University Hospital Essen, University Duisburg-Essen, 45147 Essen, Germany; 3 Department of Infectious Disease, University Hospital Essen, University Duisburg-Essen, 45147 Essen, Germany

**Keywords:** *Streptococcus pneumoniae*, vaccination, kidney transplantation, HLA antibodies, sex-specific difference

## Abstract

In transplant recipients vaccination against *Streptococcus pneumoniae* is recommended to reduce mortality from invasive pneumococcal disease. It is still debated if vaccination in transplant recipients triggers alloresponses. Therefore, it was our aim to define if vaccination with Prevenar 13^®^, a 13-valent, conjugated pneumococcal vaccine (Pfizer, New York, NY, USA) that acts T cell dependently, induces human leukocyte antigen (HLA) antibodies in clinically stable kidney transplant recipients. Forty-seven patients were vaccinated once with Prevenar 13^®^ and HLA antibodies were determined prior to vaccination and at month 1 and 12 thereafter. In parallel, pneumococcal IgG antibodies were measured. Using Luminex™ Mixed Beads technology (One Lambda/Thermo Fisher, Canoga Park, CA, USA) we observed overall no change in HLA antibodies after vaccination. Pneumococcal antibodies increased significantly at month 1 (*p* < 0.0001) and remained elevated at month 12 (*p* < 0.005). A more detailed analysis of HLA antibodies showed that in 18 females HLA class I and II antibodies increased significantly at month 1 and 12 (*p* < 0.05); whereas in 29 males HLA class I and II antibodies tended to decrease. Using Luminex™ Single Antigen Beads assay, no de novo donor-specific HLA antibodies were detected after vaccination. In conclusion, the current data indicate that females may be more susceptible to the induction of (non-specific) HLA antibodies after vaccination.

## 1. Introduction

*Streptococcus pneumoniae* (*S. pneumoniae*) is a Gram positive bacterium that frequently colonizes the human nasopharynx [1]. Outside the nasopharynx, it can cause lobar pneumonia, meningitis, otitis media or sinusitis, and it is especially harmful after coinfection with influenza virus [2]. A severe form of infection is the invasive pneumococcal diseases (IPD) which has a fatality rate of approximately 10% [1,3,4]. According to data by the Centers for Disease Control and Prevention, the rate of IPD in organ transplant recipients is 25 times greater than in the general population [3,4]. Vaccination against *S. pneumoniae* is recommended in individuals with immunocompromising conditions because it has been shown to reduce the incidence of IPD [5,6,7]. It is still under debate if vaccination may trigger alloresponses in transplant recipients [8,9,10,11,12,13,14,15,16]. Brakemeier et al. reported that after vaccination with Pandemrix, a vaccine against influenza A/H1N1 that contains potent adjuvants such as squalene, donor-specific human leukocyte antigen (HLA) antibodies were induced in kidney transplant recipients [13]. A study by Marino et al. also found a higher proportion of de novo donor-specific antibodies against HLA and major histocompatibility class I-related chain A (MICA) after influenza vaccination in kidney transplant recipients as compared to controls [15]. In contrast, a further study on kidney transplant recipients vaccinated with Pandemrix did not detect an increase of HLA and MICA antibodies after vaccination [16]. We could not observe the induction of HLA antibodies in a similar cohort vaccinated with Pneumovax, a polysaccharide vaccine against *S. pneumoniae* [14]. In contrast to immunity against influenza virus which typically involves B and T cell responses, pneumococcal capsular polysaccharides—as contained in Pneumovax—can activate B cells without T cell help, i.e., responses are T cell independent [17]. Apart from a polysaccharide vaccine against *S. pneumoniae,* there is one conjugated to a nontoxic mutant form of diphtheria toxin (Prevenar 13^®^, PCV13, Pfizer, New York, NY, USA), that acts T cell dependently. However, there is only data from a small cohort of kidney transplant recipients (*n* = 15) showing that vaccination with Prevenar did not induce human leukocyte antigen (HLA) antibodies [18]. The aim of the current study was (I) to determine in 47 kidney transplant recipients if HLA or MICA antibodies were increased after vaccination with Prevenar, using the highly sensitive Luminex™ technology, and (II) to define factors that correlate with HLA or MICA antibodies.

## 2. Materials and Methods

### 2.1. Patients

In total, 47 clinically stable kidney transplant recipients (18 female, 29 male) with a median age of 53 years (range 21–73 years) were included in this prospective, single center study (Table 1).

Stable allograft function (defined as <15% change in serum creatinine concentration within one month prior to vaccination), an interval of >3 months to kidney transplantation and absence of clinical infection, of allograft rejection and of pregnancy were defined as inclusion criteria. All patients were vaccinated with a single dose of Prevenar. During the follow-up of 12 months, four patients have received additional vaccinations (one against influenza, one against hepatitis B, one against influenza and hepatitis B and one against meningococci). Of note, seven patients (five females) were pre-sensitized against leukocyte antigens (highest panel-reactive antibodies (PRA) ≥5%) prior to transplantation. Eleven patients (nine females) received blood transfusions prior to vaccination. Heparinized blood was drawn immediately prior to vaccination and at month 1 and 12 after vaccination. The median interval between vaccination and kidney transplantation was 49 months (i.e., 4.1 years; range 4 months to 34 years). The median serum creatinine concentration (range) was 1.7 (0.9–4.9), 1.7 (1.0–5.1) and 1.6 (0.9–4.4) mg/dL pre vaccination, at month 1 and month 12, respectively, corresponding to an eGFR of 42 (11–89), 41 (11–90) and 43 (13–89) mL/min/1.73 m^2^, respectively (as determined by the Chronic Kidney Disease Epidemiology Collaboration formula [19]). Ten patients were treated with cyclosporine A, 31 with tacrolimus, 34 with mycophenolate mofetil (MMF), 44 with prednisone, four with everolimus and two with eculizumab. Thus, the majority was treated with tacrolimus, MMF and prednisone. This study was approved by the institutional review board of the University Hospital Essen (14-5858-BO) and written informed consent was obtained from all participants. It was carried out in accordance with the Declarations of Helsinki and Istanbul and its subsequent amendments.

For comparison, the sex-specificity of HLA and MICA antibodies was analyzed in a historical kidney transplant cohort from our center [14]. This cohort of 49 clinically stable kidney transplant recipients (21 female, 28 male) had a median age of 53 years (range 29–74 years) and was vaccinated once with Pneumovax. The median interval between vaccination and kidney transplantation was 6.5 years (range 5 months to 16 years) and the median serum creatinine concentration was 1.3 (0.7–4.9), 1.3 (0.6–4.9) and 1.4 (0.8–6.2) mg/dL pre vaccination, at month 1 and month 15, respectively. Twenty-six patients were treated with cyclosporin A, 18 with tacrolimus and five patients with azathioprine and MMF alone. All but three patients received prednisone. This study was also approved by the institutional review board and informed consent was obtained.

### 2.2. Vaccine

The 13-valent pneumococcal vaccine Prevenar contains polysaccharides of 13 pneumococcal serotypes, individually conjugated to a nontoxic mutant form of diphtheria toxin cross-reactive material 197 (CRM197). The vaccine contains 2.2 μg/dose of each of the serotypes, except for serotype 6B at 4.4 μg/dose. The vaccine is formulated in 5 mM succinate buffer containing 0.85% NaCl and 0.02% polysorbate 80, at pH 5.8, and contains aluminum phosphate at 0.125 mg/dose aluminum, as an adjuvant. Each 1 mL syringe contains a single 0.5 mL dose of vaccine for parenteral administration, with no preservative.

### 2.3. Determination of HLA and MICA Antibodies

All samples were tested for IgG antibodies against HLA class I, class II and MICA using Luminex™ technology-based assays (LABScreen™ Mixed Beads, One Lambda/Thermo Fisher, Canoga Park, CA, USA) according to the manufacturer’s instructions and as described in detail previously [14,20,21]. The assay enables the detection of antibodies against HLA or MICA antigens bound to fluorescently labelled polystyrene microbeads. The LABScreen™ Mixed Beads assay comprises twelve beads specific for HLA class I antibodies, five for HLA class II and two for MICA, respectively. Reactions against each bead were scored as 8 (positive), 4 (undetermined) or 1 (negative). To quantify Luminex™ reactions, we summed up the individual score values for HLA class I, HLA class II or MICA; yielding antibody score values of 12–96, 5–40 or 2–16, respectively, as published [14]. A normalized background ratio above 3 was defined as positive and between 2 and 3 as undetermined. In addition, seven patients who converted from negative to positive status for HLA class I, class II and/or MICA antibodies at month 1 after vaccination with Prevenar and six patients with rejection episodes after vacciantion were analyzed by Single Antigen Bead assay (LABScreen^TM^) according to the manufacturer’s instructions and as described previously [20,21,22].

### 2.4. Determination of Antibodies Against Pneumococci

Antibodies against *S. pneumoniae* were determined by an ELISA which detects IgG antibodies against 23 pneumococcal serotypes (VaccZyme™, The Binding Site, Schwetzingen, Germany). The assay was performed according to the manufacturer’s instructions.

### 2.5. Statistical Analysis

Data were analyzed using GraphPad Prism version 5.03 for Windows (GraphPad Prism Software, La Jolla, CA, USA) or IBM SPSS Statistics version 22 (Armonk, NY, USA). HLA and MICA antibodies prior to and post vaccination were compared by Wilcoxon matched pairs test. Time courses of antibodies in females and males were compared by 2-way ANOVA using the Bonferroni post-test. Correlation analyses of numerical variables were performed by Spearman test (two-tailed). Categorical variables were analyzed by the Mann-Whitney *U*-test. Furthermore, multinominal logistic regression was used for multivariate analysis. If not otherwise stated, mean values are indicated. Results were considered significant at *p* < 0.05.

## 3. Results

### 3.1. HLA and MICA Antibodies After Pneumococcal Vaccination

**Figure 1:**
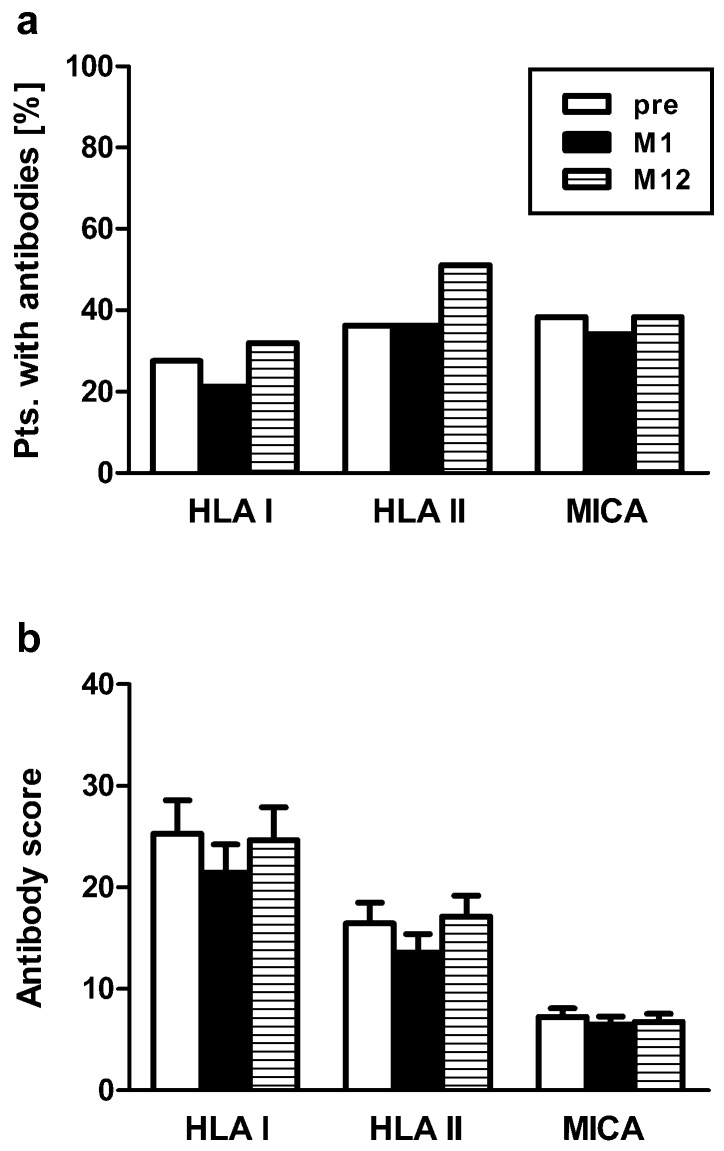
The frequency of HLA/MICA antibodies as determined by Luminex™ technology remained overall unchanged in 47 kidney transplant recipients. (**A**) shows the percentage of patients (pts.) with positive antibody responses (to at least one bead) pre vaccination and at month 1 (M1) and month 12 (M12) after vaccination with the T cell dependent pneumococcal vaccine Prevenar. Similar results were obtained when responses to individual beads were summed up and an antibody score was generated (**B**). Here, data represent mean and standard error of the mean (SEM). HLA I—HLA class I; HLA II—HLA class II; MICA—major histocompatibility class I-related chain A.

Forty-seven clinically stable kidney transplant recipients were vaccinated once with Prevenar and HLA class I and II and MICA antibodies were analyzed by Luminex™ technology (LABScreen™ Mixed Beads) prior to vaccination and at month 1 and month 12 thereafter. Positive Luminex™ reactions were present in 28%, 21% and 32% (HLA class I), 36%, 36% and 51% (HLA class II) and 38%, 34% and 38% (MICA) pre vaccination, at month 1 and 12, respectively (Figure 1A). To quantify Luminex™ reactions, we summed up the respective score values for individual Luminex™ beads. For HLA class I we obtained mean score values of 25, 21 and 25, for HLA class II 16, 14 and 17 and for MICA 7, 6 and 7, respectively (Figure 1B).

As a further step, individual courses of HLA/MICA antibodies (antibody patterns) were considered (Table 2). The majority of patients did neither display antibodies prior to vaccination nor at month 1 and 12 after vaccination, i.e., they showed the pattern Ø Ø Ø (*n* = 24 for HLA class I, *n* = 17 for HLA class II and *n* = 20 for MICA). The number of patients converting from negative antibodies prior to vaccination to positive antibodies at month 1 (shown in red) was even smaller than vice versa (shown in green). For example, four patients without HLA class I antibodies prior to vaccination showed these antibodies at month 1 and seven patients with HLA class I antibodies prior to vaccination did not show these antibodies at month 1. The quantitative analysis by antibody scores showed a similar phenomenon: in the majority of patients, the score value of HLA/MICA antibodies remained constant over time. There were fewer patients with an increase of the score value at month 1 than with a decrease (e.g., for HLA class I: 11 vs. 15). donor-specific antibodies.

Moreover, Spearman correlation analysis of antibody scores pre vaccination and at month 1 and 12 after vaccination was performed; yielding always significant, positive correlations (pre vs. month 1: *r* = 0.72, *p* < 0.0001; pre vs. month 12: *r* = 0.46, *p* = 0.001; month 1 vs. month 12: *r* = 0.49, *p* = 0.0004, data for HLA class II antibodies). Seven patients who converted from negative to positive status for HLA class I, class II and/or MICA antibodies at month 1 after vaccination, were analyzed by Single Antigen Beads assay (LABScreen^TM^, One Lambda/Thermo Fisher). None of them developed de novo.

In parallel, pneumococcal IgG antibodies were determined by ELISA (VaccZyme™); which showed a significant, 2.2-fold increase of the geometric mean titer at month 1 (*p* < 0.0001) and a 1.5-fold at month 12 (*p* < 0.005), as compared to baseline. 

### 3.2. Sex-Specific Differences in HLA Antibodies

**Figure 2:**
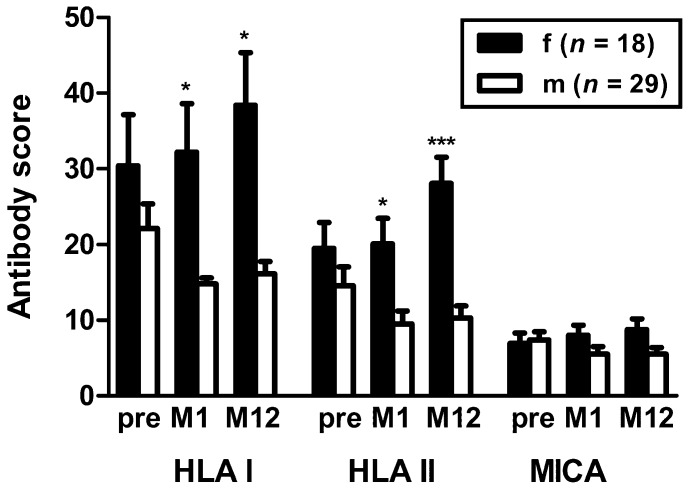
Comparison of HLA/MICA antibodies in female and male kidney transplant recipients vaccinated with Prevenar. The antibody score is the sum of responses to either HLA class I, class II or MICA Luminex™ beads. The data pre vaccination and at month 1 (M1) and month 12 (M12) after vaccination represent mean and standard error of the mean (SEM). Score values in females and males were compared by Mann-Whitney test. HLA I—HLA class I; HLA II—HLA class II; MICA—major histocompatibility class I-related chain A; * *p* < 0.05, *** *p* < 0.0001.

We analyzed if patient sex correlated with HLA or MICA antibodies. The comparison of 18 female and 29 male kidney transplant recipients-described in detail in Table 1-showed that prior to vaccination HLA class I and II antibodies were not significantly higher in females (Figure 2). However, at month 1 and 12 the difference between females and males reached statistical significance (*p* < 0.05). At month 12, this finding was even highly significant for HLA class II (*p* < 0.0001). In females HLA antibodies increased over time (HLA class II antibodies at month 12 vs. pre vaccination: *p* = 0.004); whereas in males the antibodies tended to decrease. The comparison of antibody courses in females and males by 2-way ANOVA showed that for HLA class I antibodies only the effect of sex was significant (*p* = 0.002). For HLA class II antibodies, however, sex and time (pre vaccination, month 1 and month 12) could be defined as significant factors (*p* = 0.0008 and *p* = 0.04). Moreover, the interaction between both factors was significant (*p* = 0.001). Thus, HLA class II antibodies in females may be influenced by vaccination with Prevenar. To find out if pregnancies have contributed to the higher antibody score values, female patients were asked for previous pregnancies. However, values in seven women reporting previous pregnancy did not differ significantly from those eight women without pregnancy. But score values for HLA class II antibodies pre vaccination and at month 1 thereafter tended to be higher in females with pregnancy. Antibody score values in two multiparous females did not differ from those in five females with one pregnancy. Unfortunately, data on the remaining three females were not available.

**Figure 3:**
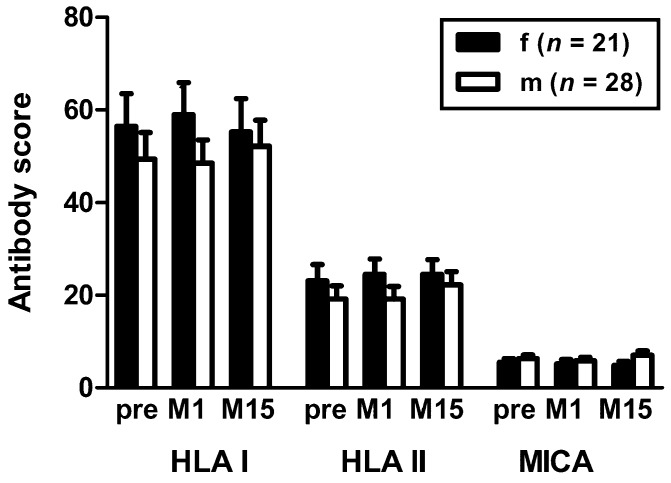
Comparison of HLA/MICA antibodies in female and male kidney transplant recipients of a historical cohort vaccinated with Pneumovax [14]. The antibody score is the sum of responses to either HLA class I, class II or MICA Luminex™ beads. The data pre vaccination and at month 1 (M1) and month 15 (M15) after vaccination represent mean and standard error of the mean (SEM). Score values in females and males did not differ significantly (Mann-Whitney test). HLA I—HLA class I; HLA II—HLA class II; MICA—major histocompatibility class I-related chain A.

To further elucidate if the different courses in females and males may be an effect of pneumococcal vaccination, we re-analyzed a historical cohort of kidney transplant recipients vaccinated with Pneumovax, a T cell independent pneumococcal vaccine [14]. In this cohort the median interval between transplantation and vaccination was 6.5 years; whereas in the current cohort the median interval was 4.1 years. Kidney transplant recipients were tested pre vaccination and at month 1 and 15 after vaccination with Pneumovax, respectively. Again, females displayed slightly higher antibody scores pre vaccination (Figure 3). At month 1 and 15 after vaccination, differences between females and males remained stable; indicating that vaccination with Pneumovax did not alter the course of HLA antibodies in females.

Moreover, after vaccination with Prevenar a larger proportion of females vs. males converted from a negative antibody status prior to vaccination to positive at month 1 thereafter (6–11% vs. 0–3%) (Table 3). On the contrary, a higher proportion of males converted from positive to negative (3–17% vs. 0–6%). Similar findings were observed when considering the change of the sum score. Females were over-represented in the group that showed an increase within one month after vaccination; whereas males were over-represented in the group with a decrease.

In one female, C4d negative, antibody-mediated rejection was newly diagnosed at month 15 after vaccination with Prevenar (#1 in Table 4). This 25-year old female had received her first graft 10 years prior to vaccination from a female, unrelated living donor. She had not been pregnant and had not received blood transfusions. Donor-specific HLA class II antibodies (against HLA-DQ3, median fluorescence intensity (MFI) of 9700) were observed already 11 months prior to vaccination and decreased after vaccination (MFI of 1200). But score values of HLA antibodies increased after vaccination (HLA class I: 12, 24 and 24; HLA class II: 36, 40 and 40; values pre vaccination and at month 1 and 12 after vaccination). In parallel, serum creatinine concentrations increased from 1.8–2.1 and 2.9 mg/dL, respectively. After the diagnosis of antibody-mediated rejection, three plasma separations were performed and the patient received intravenous IgG (cumulative dose of 60 g). Nevertheless, allograft function was lost at month 32 after vaccination. At month 40 she received a kidney graft from her mother.

Four further females suffered from antibody-mediated rejection before and after vaccination (at month 2, 7, 8 and 21) (#2 to #5 in Table 4). It was classified in all cases as chronification of antibody-mediated rejection. The first patient, a 27-year old female transplanted in 2011, had a PRA value of 30% prior to transplantation and prior to vaccination which decreased to 25% after vaccination (#2). Luminex™ Single Antigen Beads testing showed a decrease of HLA antibody specificities after vaccination and the absence of donor-specific HLA antibodies. In the second patient (25-year old), the PRA value was <5% prior to and post vaccination (#3). Luminex™ Single Antigen Beads testing revealed HLA class I and II antibodies. Again, the HLA antibody specificities decreased after vaccination and no de novo donor-specific HLA antibodies were found. This patient has been transplanted 2011 in Iraq and, unfortunately, no data on the deceased donor are available. Therefore, it remains unclear if the HLA antibodies found pre- and post-vaccination are donor-specific. The third patient, a 66-year old female, was transplanted in 2010 and 2012 and had a PRA of 5% prior to the second transplantation, but was PRA negative prior to and post vaccination (#4). Luminex™ testing revealed donor-specific HLA class I and II antibodies against the first (but not the second) graft pre and post vaccination (MFI values for HLA-A2: stable with 2200; HLA-DR7: 17.000 and 10.700; HLA-DR9: 11500 and 8000; HLA-DQ2: 12.500 and 9300). Thus, MFI values remained stable or decreased after vaccination. The fourth patient, a 53-year old female transplanted in 1997 and 2006, had a PRA of 20% prior to the second transplantation, but was PRA negative prior to and post vaccination (#5). Luminex™ testing showed donor-specific antibodies against the first graft (MFI values for HLA-B8: 14.300 and 17.900; HLA-Cw7: 3900 and undetectable) and against both grafts (MFI values for HLA-DR51: 2000 and 1300; HLA-DQ6: 900 and 600). Thus, overall donor-specific antibodies remained stable also in this forth female. But at month 12 after vaccination, antibody scores for HLA class I and II increased. Two out of these four patients with pre-existing antibody-mediated rejection lost their graft and one of these two died due to pneumonia with sepsis. In addition, one of the patients without graft loss died due to septicemia and multi organ failure. In summary, in none of these four patients de novo donor-specific antibodies could be detected after vaccination.

**Figure 4:**
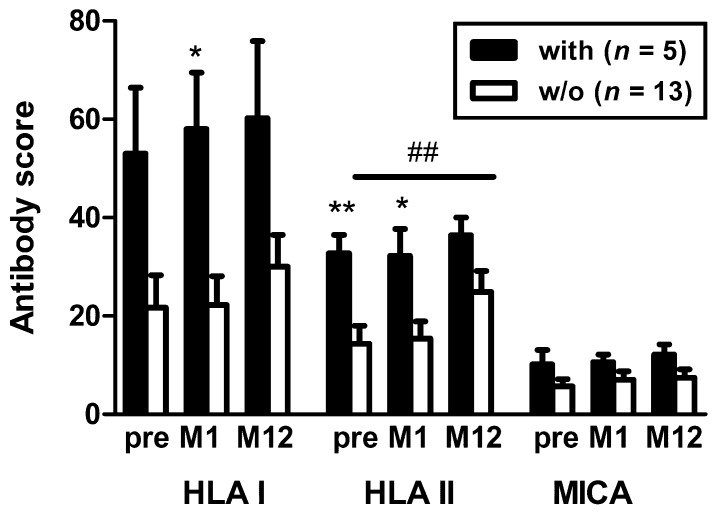
Comparison of HLA/MICA antibodies in females with and without antibody-mediated rejection after vaccination with Prevenar. Antibodies were considered pre vaccination and at month 1 (M1) and month 12 (M12) thereafter. Data represent mean and standard error of the mean (SEM). The antibody score is the sum of responses to either HLA class I, class II or MICA Luminex™ beads. Score values for HLA and MICA antibodies in females with vs. without rejection were higher (* *p* < 0.05, ** *p* < 0.005, Mann-Whitney test). Score values for HLA class II antibodies in females without rejection were significantly higher at month 12 vs. pre vaccination (^##^ *p* = 0.008, Wilcoxon matched pairs test). HLA I—HLA class I; HLA II—HLA class II; MICA—major histocompatibility class I-related chain A; w/o—without.

As expected, sum scores for HLA class I and II antibodies in five females with vs. 13 without antibody-mediated rejection were significantly (*p* < 0.05) higher (Figure 4). Interestingly, in females without rejection score values increased over time; reaching statistical significance (*p* = 0.008) for HLA class II antibodies at month 12 vs. pre-vaccination.

None of the males suffered from antibody-mediated rejection. Only one male patient showed rejection (at month 2) after vaccination, which was classified as cellular borderline rejection, presumably linked to the ABO incompatible transplantation. Of note, none of the characteristics at the time of vaccination differed significantly between females and males (Table 1). But females tended to have more panel-reactive antibodies at the time of transplantation; indicating a higher rate of pre-sensitization against HLA.

An analysis of the course of antibodies was performed also in the historical cohort vaccinated with Pneumovax (Table 5). As in the current cohort, more females were positive for HLA class I and class II antibodies at all three time points (pattern + + +). Apart from that finding, antibody patterns and courses were similar in females and males.

### 3.3. Correlation of Additional Clinical Parameters and Antibodies

**Figure 5:**
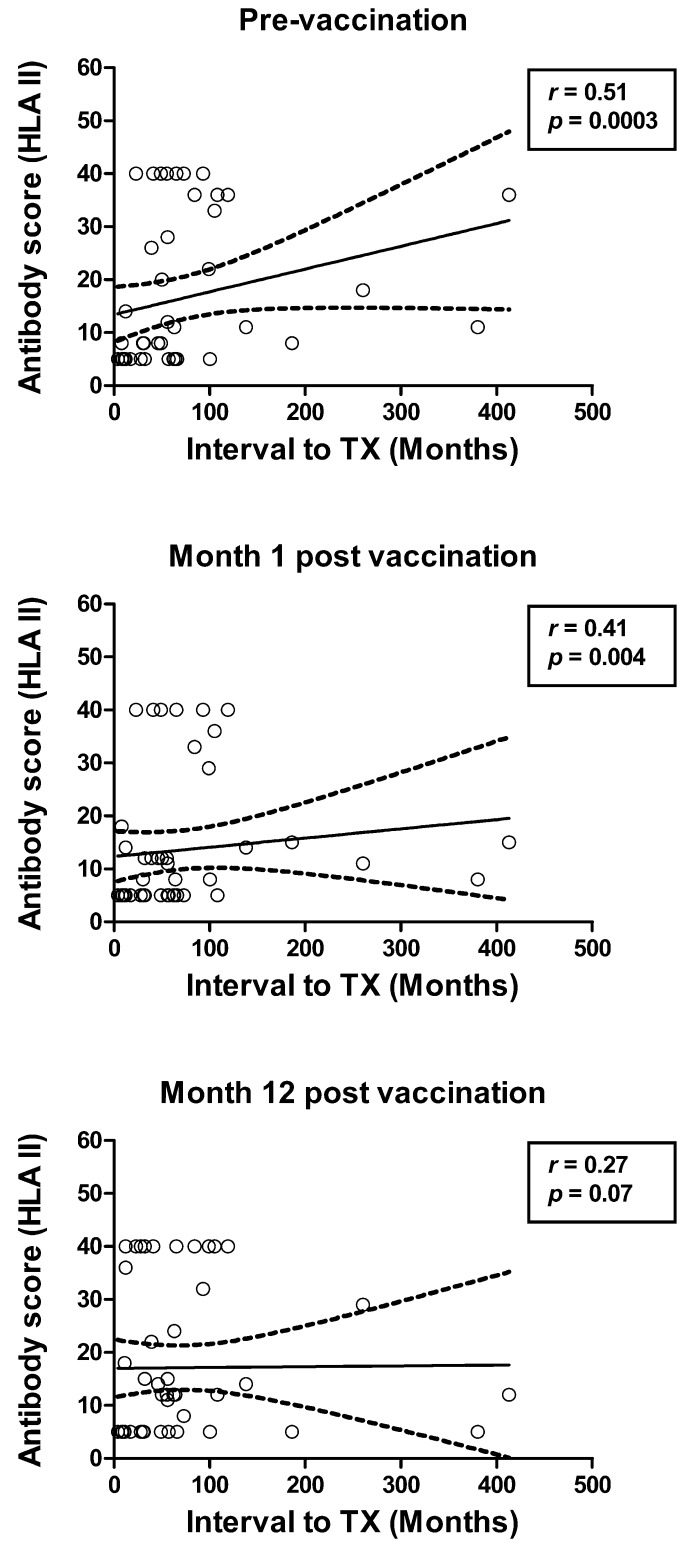
Spearman correlation analysis of the interval between kidney transplantation (TX) and vaccination and HLA class II (HLA II) antibodies. The antibody score is the sum of responses to HLA class II Luminex™ Mixed beads. Correlation analyses were performed two-tailed prior to and at month 1 and 12 after vaccination with Prevenar (*n* = 47). The continuous line represents the regression line, the broken lines the 95% confidence interval.

It was analyzed if age, kidney function (serum creatinine concentration or estimated glomerular filtration rate), albuminuria, interval between transplantation and vaccination, concentration of pneumococcal antibodies, previous pneumococcal vaccination, other vaccinations, presence of panel-reactive antibodies, blood transfusions, rejection, re-transplantation and immunosuppressive regimen correlated with HLA or MICA antibodies. Spearman analysis indicated that the only numerical variable correlating significantly with HLA or MICA antibody scores was the interval between transplantation and vaccination (Table 6 and Figure 5). The correlation coefficient was always positive and the highest values were obtained for HLA class II antibodies (Figure 5). Concerning kidney function, there were two patients with an increase of the serum creatinine concentration of >20% after vaccination. One of them had HLA class I antibodies prior to vaccination, the other one had HLA class I and II antibodies prior to vaccination. In the first patient, the HLA antibodies even disappeared at month 1 after vaccination, and in the second, the HLA class I antibodies remained constant and the HLA class II and MICA antibodies decreased.

The analysis of categorical variables by Mann-Whitney test showed that seven patients with panel-reactive antibodies prior to transplantation (maximum PRA ≥ 5%) had significantly higher HLA antibody scores than those with negative PRA (Table 7). This was observed prior to and post vaccination. Eleven patients with vs. without transfusions prior to vaccination had higher antibody scores during the whole study period. However, this finding only reached statistical significance for antibodies against HLA class II at month 12 (*p* = 0.02). In six patients with subsequent antibody-mediated or cellular allograft rejection sum scores for HLA class I and II antibodies were significantly higher (*p* < 0.05). This also accounts for values prior to and post vaccination. More detailed data on the female patients with antibody-mediated rejection are given in the previous chapter. Similarly, in six patients with prior transplantation antibody scores tended to be higher pre and post vaccination. One female and one male who received vaccination against influenza-either six months after vaccination against pneumococci or simultaneously—showed an increase of HLA class I and II antibody scores at month 12, but the two patients vaccinated against hepatitis B (only) or against meningococci displayed no change of the antibody scores. Previous pneumococcal vaccination and the immunosuppressive drug regimen did not correlate with HLA or MICA antibody scores.

In summary, sex, pre-sensitization (as indicated by a PRA ≥ 5%), prior transfusion, allograft rejection and interval between transplantation and vaccination appear as important factors correlating with HLA and MICA antibody scores. As females and males differed non-significantly in their rate of PRA positivity and significantly (*p* < 0.0001) in their rate of previous transfusions we included these two factors together with the patient sex into multivariate analysis. Patient sex could be identified as an independent factor correlating with HLA class I and II antibody scores at month 12 after vaccination (*p* = 0.0006 and *p* =0.049, respectively).

## 4. Discussion

The current study shows that there is no evidence for an induction of donor-specific HLA antibodies after vaccination with Prevenar. This finding is in line with a recent study on 15 kidney transplant recipients that did not show de novo HLA antibodies after vaccination with this pneumococcal vaccine [18]. However, in our study the time course of HLA class II antibodies differed significantly between female and male patients after receiving the T cell dependent vaccine Prevenar. It is well established that pre-transplant HLA antibodies are more abundant in women due to previous pregnancy [23,24]. This can lead, for example, to higher donor-specific reactions in flow cytometric crossmatches of female kidney transplant recipients [22]. It is expected that also after transplantation HLA antibodies could be observed more frequently in females. But there is one study showing almost equal frequencies of MICA antibodies in females and males prior to kidney transplantation [25]. Similarly, in the current study HLA antibodies differed between females and males, but MICA antibodies did not. We are not aware of any data showing that vaccination may have an effect on HLA antibodies which presents differently in females and males. To further address this question, we re-analyzed historical data of kidney transplant recipients vaccinated with Pneumovax, a T cell independent pneumococcal vaccine [14]. Interestingly, in this cohort the time course of HLA antibodies after vaccination was not influenced by the patient sex.

According to the current literature, immune responses in females and males differ, e.g., in the concentration of cytokines or in antibodies produced after certain vaccinations [26,27,28,29,30,31,32,33]. For example, in females cytomegalovirus pp65-specific IL-21 ELISpot responses were higher [30] or antibody titers after vaccination against hepatitis B virus were increased [26]. A recent study showed that vaccine-induced pneumococcal IgG levels were slightly higher in girls, but only between the primary series and the 11-month booster [32]. This study described T cell dependent pneumococcal vaccines (PCV7/PCV10/PCV13). Another study described higher specific antibody concentrations after applying pneumococcal polysaccharide vaccines in female children between 6 and 9 years, no difference in adults and lower concentrations in aged females (>65 years) [33]. Pneumococcal antibodies as determined in the current cohort and also in the previous study with Pneumovax did not differ significantly between females and males. But in both cohorts (median age 53 years) responses in males tended to be higher; which is in accordance with data on aged vaccinees [33]. In addition, a recent review described that in adult and also aged females T cell activation and proliferation as well as B cell numbers were increased [31]. As several immune responses are sex-specific, it is possible that females respond differently to the T cell dependent vaccine Prevenar. Stimulation of the T cell receptor by vaccine antigens may induce by cross-reactivity T cell help for the induction HLA antibodies. For herpes viruses, this cross-reactivity is known for many years. For example, in 1997 Burrows et al. already described cross-reactive memory T cells for the Epstein-Barr virus which augmented the alloresponse to common HLA molecules [34].

In contrast to Prevenar, Pneumovax stimulates only B cells as it contains capsular polysaccharide antigens that cross-link B cell receptor molecules [17]. Due to the lack of T cell involvement, it may be less likely that Pneumovax induces HLA antibodies. There are only few studies addressing a possible link between pneumococcal vaccination and rejection in kidney transplant recipients [14,18,35,36]. None of these studies described episodes of rejection or adverse reactions to vaccination with a T cell independent (polysaccharide) vaccine [14,35,36] or a T cell dependent (conjugated) vaccine [18,35]. In the current study, in one female antibody-mediated rejection was newly diagnosed at month 15 after vaccination. Four further females with pre-existing antibody-mediated rejection showed another episode of rejection. It is difficult to assess if vaccination may have caused or triggered rejection. However, in none of the females de novo donor-specific HLA antibodies were observed after vaccination and the MFI values of pre-existing donor-specific HLA antibodies remained constant or even decreased. Thus, a causal relation appears as unlikely but cannot be excluded with certainty. A previous publication described de novo donor-specific HLA antibodies in 6 out of 107 renal transplant patients receiving Pandemrix, an adjuvanted vaccine against influenza A/H1N1 which stimulates B and T cell responses [13]. This finding is supported by one further study on influenza vaccination of kidney transplant recipients [15] but unsupported by another one [16]. In our current study the two patients who received influenza vaccination showed an increase of their HLA antibody scores. However, two patients are too few to draw any valid conclusion. Marino et al. [15] suggested that the development of de novo anti-HLA and anti-MICA antibodies after an external stimulus other than HLA antigens is possible. Pandemrix contains the highly potent adjuvant AS03 (squalene, D,L-α-tocopherol and polysorbate 80). It has been shown that AS03 induced a 10-fold higher antibody response than aluminium hydroxide two weeks after HBsAg vaccination and that IL-6, CXCL1, CSF3, CCL2, CCL3 and CCL5 were increased after vaccination; leading to the recruitment of monocytes and dendritic cells and the induction of NF-κB [37]. Moreover, squalene augmented antigen-specific Th1 responses in vaccinated mice through a caspase/IL-18-dependent mechanism [38]. It is possible that the adjuvant AS03 contributed to the increase of donor-specific HLA antibodies after vaccination. However, Prevenar does neither contain AS03 nor squalene. Moreover, as shown by Locke et al. [39] proinflammatory conditions such as infection, minor surgeries, major medical events (e.g., myocardial infarction) and traumatic injury could lead to an increase in HLA antibodies.

It is well established that sensitization against HLA and MICA prior transplantation adversely impacts on the graft outcome [25,40,41]. Tallying with this, in the current study the five patients who lost their graft or two who died tended to have higher antibody scores. In patients with vs. without sensitization prior to transplantation antibody scores pre and post vaccination remained significantly higher. Similarly, patients receiving transfusions displayed higher HLA antibody scores.

In addition, we observed that the interval between transplantation and vaccination correlated positively with higher antibody scores. However, this correlation is based on only a few datasets modifying the curve as shown in Figure 5. Nevertheless, this finding is in line with publications showing that the frequency of HLA antibodies increases after transplantation; which leads to chronic antibody-mediated rejection and allograft failure in the long-term [40,42,43,44].

## 5. Conclusions

This study is the first showing that female kidney transplant recipients may be more susceptible to the induction of HLA antibodies after vaccination with Prevenar. Fortunately, however, these HLA antibodies were not donor-specific. In addition, most likely due to pregnancies, HLA antibodies were observed more frequently in female transplant recipients. Thus, allo-sensitization may be further augmented by vaccination. A deeper understanding of the effect of sex and pregnancy on immune responses might help to optimize vaccination programmes (e.g., timing or reduced dose of the vaccine) in order to ensure a sufficient strength of immune responses and minimize adverse reactions, as suggested by a previous publication [45]. Especially in sensitive groups such as transplant recipients the sex-specificity of vaccination responses should be considered.

## Figures and Tables

**Table 1:** Characteristics of 47 kidney transplant recipients at the time of vaccination with Prevenar.

Parameter	Females (*n* = 18)	Males (*n* = 29)
Median age (range), years ^1^	52 (25–67)	57 (21–73)
Median interval TX-vaccination (range), months	52 (9–260)	49 (4–413)
Donor sex (female/male/unknown ^2^)	9/8/1	12/17/0
Deceased/living donor	13/5	24/5
Relation between recipient and living donor		
Brother/Sister	1	1
Father/Mother	1	1
Spouse/Partner	2	2
Other	1	1
HLA mismatches ^2,3^ (mean ± SD)	2.3 ± 1.9	2.3 ± 1.8
HLA antibody status prior to TX		
Current PRA, no.		
<5%	14	27
≥5% [values]	3 [5; 20; 30%]	2 [10; 20%]
Unknown ^2^	1	0
Highest PRA, no.		
<5%	12	27
≥5% [values]	5 [6; 15; 34; 60; 70%]	2 [10; 20%]
Unknown ^2^	1	0
Cold ischemic time ^2^		
(hours, mean ± SD)	11.5 ± 7.4	11.3 ± 7.9
Median serum creatinine (range), mg/dl		
Pre vaccination	1.7 (0.9–3.4)	1.7 (1.2–4.9)
Month 1 post vaccination	1.8 (1.0–3.3)	1.6 (1.0–5.1)
Month 12 post vaccination	1.8 (0.9–4.4)	1.6 (1.0–3.0)
Median eGFR (range), ml/min/1.73 m^2^		
Pre vaccination	42 (25–84)	37 (11–89)
Month 1 post vaccination	42 (20–78)	36 (11–90)
Month 12 post vaccination	45 (19–89)	41 (13–85)
Immunosuppression, no. ^1^		
CsA + MMF + CS	4	5
CsA + CS	1	0
TAC + MMF + CS	6	12
TAC + ECU + CS	2	0
TAC + EVR + CS	0	1
TAC + MMF	1	1
TAC + CS	1	7
AZA + CS	0	1
MMF + EVR + CS	1	2
MMF + BEL + CS	1	0
MMF only	1	0
Kidney transplantation, no.		
1st	15	26
2nd	3	2
3rd	0	1
Rejections after vaccination, no. ^4^		
Antibody-mediated rejection ^5^	1 ^6^	0
Cellular ^5^	0	1 ^7^
Allograft loss, no. ^4^	3	2

^1^ At the time of vaccination; ^2^ one female patient was transplanted in May 2011 in Iraq and no data on the panel-reactive antibodies (PRA) or the deceased donor are available; ^3^ HLA mismatches in host-vs-graft (HvG) direction defined by low resolution HLA-A, B, DRB1 typing; ^4^ within a median follow-up of 51 months (range 44–57) after vaccination; ^5^ newly diagnosed rejections after vaccination; ^6^ antibody-mediated rejection occurring at month 15 after vaccination; ^6^ cellular rejection occurring at month 2 after vaccination; AZA—azathioprine; BEL—belatacept; CS—corticosteroids; CsA—cyclosporine A; ECU—eculizumab; eGFR—estimated glomerular filtration rate; EVR—everolimus; MMF—mycofenolate mofetil; ND—not determined; TAC—tacrolimus; TX—transplantation; Differences between females and male were analyzed by Mann-Whitney test or Fisher’s exact test as appropriate. None of the parameters differed significantly.

**Table 2:** Individual courses of HLA/MICA antibodies in 47 kidney transplant recipients pre and at month 1 (M1) and month 12 (M12) after vaccination with Prevenar.

**Antibody Pattern**			**n Patients with This Pattern**		
**pre**	**M1**	**M12**	**HLA I**	**HLA II**	**MICA**
ø	ø	ø	24	17	20
ø	ø	+	6	8	7
ø	+	ø	2	3	1
ø	+	+	2	2	1
+	ø	ø	5	2	3
+	ø	+	2	3	1
+	+	ø	1	1	5
+	+	+	5	11	9
**Antibody score pre-M1**			**HLA I**	**HLA II**	**MICA**
↑			11	10	6
=			21	23	26
↓			15	14	15

HLA I—HLA class I; HLA II—HLA class II; MICA—major histocompatibility class I-related chain A; score—sum of reactions towards Luminex™ Mixed Beads; Ø—negative; +—positive; ↑—increase; =—identical; ↓—decrease.

**Table 3:** Individual courses of HLA/MICA antibodies in 18 female and 29 male kidney transplant recipients pre and at month 1 (M1) and month 12 (M12) after vaccination with Prevenar.

**Antibody Pattern**			**% of Females with This Pattern**			**% of Males with This Pattern**		
**pre**	**M1**	**M12**	**HLA I**	**HLA II**	**MICA**	**HLA I**	**HLA II**	**MICA**
ø	ø	ø	28	11	33	66	52	48
ø	ø	+	22	22	17	7	14	14
ø	+	ø	6	11	6	3	3	0
ø	+	+	11	6	6	0	3	0
+	ø	ø	0	0	6	17	7	7
+	ø	+	6	6	0	3	7	3
+	+	ø	6	0	6	0	3	14
+	+	+	22	44	28	3	10	14
**Antibody score pre - M1**			**HLA I**	**HLA II**	**MICA**	**HLA I**	**HLA II**	**MICA**
↑			39	39	33	14	10	0
=			50	44	50	41	52	59
↓			11	17	17	45	38	41

HLA I—HLA class I; HLA II—HLA class II; MICA—major histocompatibility class I-related chain A; score—sum of reactions towards Luminex™ Mixed Beads; Ø—negative; +—positive; ↑—increase; =—identical; ↓—decrease.

**Table 4:** Course of HLA/MICA antibodies in five female kidney transplant recipients with antibody-mediated rejection after vaccination with Prevenar.

ID	HLA I			HLA II			MICA		
	pre	M1	M12	pre	M1	M12	pre	M1	M12
#1	12	24	24	36	40	40	2	8	12
#2	96	96	96	26	12	22	16	9	16
#3	46	53	28	40	40	40	12	12	5
#4	57	60	57	40	40	40	5	8	12
#5	54	57	96	22	29	40	16	16	16

The antibody score (sum of reactions towards Luminex™ Mixed Beads) was determined pre vaccination and at month 1 (M1) and month 12 (M12) after vaccination. HLA I—HLA class I; HLA II—class II; MICA—major histocompatibility class I-related chain A; #—patient number.

**Table 5:** Individual courses of HLA/MICA antibodies in 21 female and 28 male kidney transplant recipients pre and at month 1 (M1) and month 15 (M15) after vaccination with Pneumovax.

**Antibody Pattern**			**% of Females with This Pattern**			**% of Males with This Pattern**		
**pre**	**M1**	**M12**	**HLA I**	**HLA II**	**MICA**	**HLA I**	**HLA II**	**MICA**
ø	ø	ø	28	33	72	21	45	55
ø	ø	+	0	6	0	3	14	10
ø	+	ø	17	6	0	3	0	3
ø	+	+	0	0	11	7	3	0
+	ø	ø	0	6	6	3	0	3
+	ø	+	11	0	6	3	3	0
+	+	ø	0	6	6	7	0	7
+	+	+	61	61	17	48	31	17
**Antibody score pre - M1**			**HLA I**	**HLA II**	**MICA**	**HLA I**	**HLA II**	**MICA**
↑			22	33	17	41	21	17
=			56	67	83	28	55	55
↓			39	17	17	28	21	24

HLA I—HLA class I; HLA II—HLA class II; MICA—major histocompatibility class I-related chain A; score—sum of reactions towards Luminex™ Mixed Beads; Ø—negative; +—positive; ↑—increase; =—identical; ↓—decrease.

**Table 6:** Correlation of interval between transplantation and vaccination and HLA/MICA antibodies in 47 kidney transplant recipients pre and at month 1 (M1) and month 12 (M12) after vaccination with Prevenar.

Antibody Specificity	*r*	*p*
HLA class I		
Pre	0.26	0.08
M1	0.31 *	0.03
M12	0.19	0.19
HLA class II		
Pre	0.51 *	0.0003
M1	0.41 *	0.004
M12	0.27	0.07
MICA		
Pre	0.25	0.09
M1	0.30 *	0.04
M12	0.14	0.35

For the Spearman correlation analysis, antibody scores (sum of reactions towards Luminex™ Mixed Beads) were considered. MICA—major histocompatibility class I-related chain A; *r*—correlation coefficient; *—significant result.

**Table 7:** Comparison of kidney transplant recipients with and without panel-reactive antibodies (maximum PRA < 5%) prior to transplantation. Antibody scores were analyzed pre-vaccination and at month 1 (M1) and month 12 (M12) after vaccination with Prevenar.

**Antibody Specificity**	**PRA ≥ 5% (*n* = 7)**	**PRA < 5% (*n* = 39)**	***p***
**HLA class I**			
Pre	53.7 *	20.5	0.01
M1	46.0 *	16.8	0.04
M12	50.9	20.3	0.053
**HLA class II**			
Pre	29.3 *	14.4	0.02
M1	24.9 *	11.5	0.048
M12	28.4	15.4	0.07
**MICA**			
Pre	11.3	6.6	0.052
M1	9.7	6.0	0.1
M12	10.9	6.2	0.08

The table shows mean values for antibody scores (sum of reactions towards Luminex™ Mixed Beads) and the significance of differences as determined by Mann-Whitney test. PRA data in one patient were not available. MICA—major histocompatibility class I-related chain A; *—significant result.
